# Observational Study of a French and Belgian Multicenter Cohort of 23 Patients Diagnosed in Adulthood With Mevalonate Kinase Deficiency

**DOI:** 10.1097/MD.0000000000003027

**Published:** 2016-03-18

**Authors:** Cécile-Audrey Durel, Achille Aouba, Boris Bienvenu, Samuel Deshayes, Brigitte Coppéré, Bruno Gombert, Cécile Acquaviva-Bourdain, Eric Hachulla, Frédéric Lecomte, Isabelle Touitou, Jacques Ninet, Jean-Baptiste Philit, Laurent Messer, Marc Brouillard, Marie-Hélène Girard-Madoux, Michel Moutschen, Nadia Raison-Peyron, Pascal Hutin, Pierre Duffau, Pierre Trolliet, Pierre-Yves Hatron, Philippe Heudier, Ramiro Cevallos, Thierry Lequerré, Valentine Brousse, Vincent Lesire, Sylvain Audia, Delphine Maucort-Boulch, Laurence Cuisset, Arnaud Hot

**Affiliations:** From the Internal Medicine Department, Edouard Herriot Hospital, Lyon (C-AD, BC, JN, M-HG-M, AH); Internal Medicine Department, Côte de Nacre Hospital, Caen (AA, BB, SD); Medicine and Rheumatology Department, Saint-Louis Hospital, La Rochelle (BG); Inborn Errors of Metabolism Laboratory, Civil Hospital of Lyon, Bron (CA-B); Internal Medicine Department, Claude Huriez Hospital, Lille (EH, P-YH); Polyvalent Medicine Department, Cornouaille Hospital Center, Quimper (FL, PH); Autoinflammatory Diseases Medical Unit, Arnaud Villeuneuve Hospital, Montpellier (IT); Nephrology Department, Metropole Savoie Hospital Center, Chambéry (J-BP); Rheumatology Department, Louis Pasteur Hospital, Colmar (LM); Hematology Department, Arras Hospital Center, Arras, France (MB); Internal Medicine Department, Sart Tilman, Liège, Belgique (MM); Dermatology and Allergology Department, Saint-Eloi Hospital, Montpellier (NR-P); Internal Medicine Department, Saint-André Hospital, Bordeaux (PD); Nephrology Department, Lyon Sud Hospital Center, Pierre-Bénite (PT); Hematology Department, Princesse Grace Hospital Center, Monaco (PH); Internal Medicine Department, Saint-Vincent Hospital Center, Strasbourg (RC); Rheumatology Department, Charles Nicole Hospital, Rouen (TL); Department of Pediatrics, Necker-Enfants Malades Hospital, Paris (VB); Diabetology and Internal Medicine Department, Blois Hospital Center, Blois (VL); Internal Medicine Department, Bocage Central, Dijon (SA); Service de Biostatistique, Hospices civiles de Lyon, Université de Lyon 1, Villeurbanne; CNRS, UMR5558, Laboratoire de Biométrie et Biologie Evolutive, Equipe Biostatistique-Santé, Villeurbanne (DM-B); and Department of Biochemical Genetics, Hospital and Institut Cochin, Paris (LC), France.

## Abstract

The aim of this study was to describe the clinical and biological features of Mevalonate kinase deficiency (MKD) in patients diagnosed in adulthood.

This is a French and Belgian observational retrospective study from 2000 to 2014. To constitute the cohort, we cross-check the genetic and biochemical databases. The clinical, enzymatic, and genetic data were gathered from medical records.

Twenty-three patients were analyzed. The mean age at diagnosis was 40 years, with a mean age at onset of symptoms of 3 years. All symptomatic patients had fever. Febrile attacks were mostly associated with arthralgia (90.9%); lymphadenopathy, abdominal pain, and skin lesions (86.4%); pharyngitis (63.6%); cough (59.1%); diarrhea, and hepatosplenomegaly (50.0%). Seven patients had psychiatric symptoms (31.8%). One patient developed recurrent seizures. Three patients experienced renal involvement (13.6%). Two patients had angiomyolipoma (9.1%). All but one tested patients had elevated serum immunoglobulin (Ig) D level. Twenty-one patients had genetic diagnosis; most of them were compound heterozygote (76.2%). p.Val377Ile was the most prevalent mutation. Structural articular damages and systemic AA amyloidosis were the 2 most serious complications. More than 65% of patients displayed decrease in severity and frequency of attacks with increasing age, but only 35% achieved remission.

MKD diagnosed in adulthood shared clinical and genetic features with classical pediatric disease. An elevated IgD concentration is a good marker for MKD in adults. Despite a decrease of severity and frequency of attacks with age, only one-third of patients achieved spontaneous remission.

## INTRODUCTION

Mevalonate kinase deficiency (MKD) is a rare autoinflammatory autosomal recessive periodic fever disorder.^1,2^ Hyperimmunoglobulinemia D and periodic fever syndrome (HIDS) and mevalonic aciduria (MA) are both part of the MKD spectrum.^3,4^ They result from mutations in the gene encoding mevalonate kinase (MK). This is a key enzyme in cholesterol and non-sterol isoprenoids biosynthesis, which are important compounds in a lot of vital cellular functions. A deficient MK activity leads to shortage of isoprenoids downstream compounds and systemic inflammation through interleukin (IL)-1β-mediation.^5^ HIDS and MA are the 2 extreme phenotypes of a continuous spectrum with a mild and severe form, respectively, correlated with the residual MK activity.^6–9^ In fact, there must be apparently healthy people to stillbirth.^10^ The patients suffered from lifelong febrile attacks that last 3 to 7 days and recur every 4 to 6 weeks with varying associated combination symptoms, such as painful lymphadenopathy, abdominal pain, arthralgia or arthritis, diarrhea and vomiting, skin lesions, headache, cold chills, aphtous ulcers, and hepatosplenomegaly.^11^ MA patients had also dysmorphic features, growth retardation, and neurological involvement.

HIDS is the historical name of the pathology and owes its name to the increase of immunoglobulin (Ig) D serum level reported in the first description.^7^ Nevertheless, elevated IgD remains an inconsistent finding.^12,13^

Despite a mean diagnostic delay >10 years, first HIDS attacks usually occur in early childhood.^12,14^ Exceptional worldwide cases of adult-onset MKD without previous feverish history have been reported but poorly described.^14^ We conduct this study to assess the clinical and biological conditions of a multicenter cohort of patients diagnosed with MKD in adulthood. We describe the spectrum of clinical and biological phenotype, and genotype at diagnosis. The long-term complications of the spontaneous course of the disease are noted.

## METHOD

Patients were identified on genetic analysis, or enzymatically proven with a family history of MKD or typical clinical features, in France and Belgian. This was a retrospective study from January 2000 (after the availability of the genetic assay) to December 2014.^3^ To constitute an exhaustive cohort, we cross-checked the database of the 2 French competent genetic laboratories in Paris and Montpellier (Drs Cuisset and Touitou) and those of the French specialized biochemical laboratory in Lyon (Dr Acquaviva-Bourdain). The genomic DNA was extracted from primary skin fibroblasts, white blood cells (ie, lymphocytes and leukocytes), or lymphoblasts from MKD patients.

The inclusion criteria were: age upon 16 years at diagnosis; presence of 2 pathological mutations in the MK gene; or low lymphocyte and/or fibroblast MK activity, and/or increased MA in patients with genetically MKD family history and/or typical recurrent febrile attacks (without individual molecular diagnosis). The patients with insufficient collected data were excluded. The data were recorded from the completed questionnaire sent with the enzymatic analysis request, and from the contact with all referring physicians (e-mail and/or phone). The authorizations from physicians and patients were requested. We collected demographic data (gender, age at diagnosis, age at onset of disease, family history, vital status), clinical features (length and recurrence of febrile crises, evolution of crises with increasing age, triggering factors, organ involvement), biological evaluation (inflammatory markers, serum Ig concentrations, MK activity, MA, genetic features), histological results if available, and tried treatments after diagnosis. The alternative diagnoses evoked before MKD diagnosis was recorded. The remission was defined as the absence of both clinical symptoms and inflammatory biological syndrome.

## RESULTS

### Demographic Features

Twenty-seven patients were screened. Four patients were excluded: diagnosis before 16 years in 1 patient, and insufficient data in 3 patients. Twenty-three patients (14 women and 9 men) were analyzed, including 1 asymptomatic patient. Some patients have been previously described in case reports or series.^12,14,15^

The demographic data were shown in Table 1. The mean follow-up after diagnosis was 7 years (range 1–13). The mean age at diagnosis was 40 years (range 16–79), with a mean age at onset of symptoms of 9 years (range 0.2–77). A patient had a late onset of manifestations at age of 77 years with a diagnosis made 2 years later. The diagnosis was delayed with a median of 30.6 years after onset of symptoms. Ten patients (43.5%) had familial histories of MKD. Thirteen patients (59.1%) had first clinical features before 5 years, and all but 4 before 10 years (81.8%). At time of diagnosis, 9 patients (40.9%) had a mean of 1 to 2 attacks monthly, and 11 (50%) had ≤1 monthly. Triggering factors were identified in 16 patients (72.7%), which were infection (n = 12), vaccination (n = 9), and condition associated with estrogen level variation as pregnancy in 3 patients and menstrual period in one patient.

**TABLE 1:**
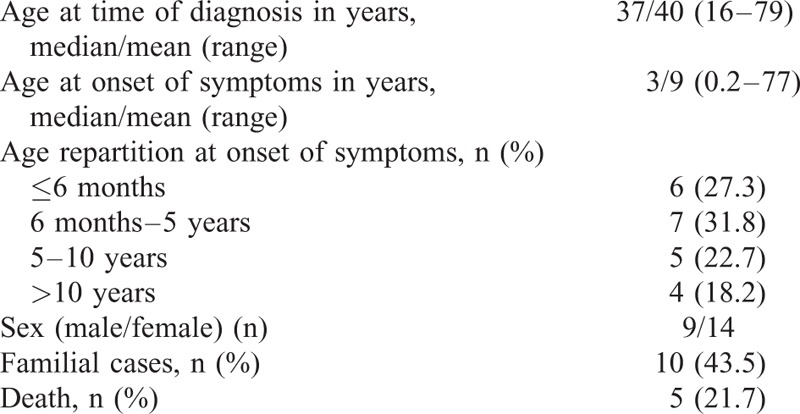
Demographic Data

Most of the time, febrile attacks were triggered by viral ear-nose-throat infections.

Fourteen patients (60.7%) had an alternative diagnosis before diagnosis of MKD: 9 juvenile idiopathic arthritis, 3 rheumatoid purpura, and 2 acute articular rheumatisms.

### Clinical Features

Table 2 showed the main clinical features. Considering the symptomatic patients, febrile attacks were noticed in all patients. The median duration of febrile attacks ranged from 3 to 7 days, never under 2 days or upon 10 days. The articular symptoms affected 21 patients (95.5%), being most of time arthralgia without synovitis (90.9%). One patient developed severe destructive arthritis mimicking rheumatoid arthritis. A tumoral syndrome occurred in 21 patients (95.5%). Nineteen patients had lymphadenopathies (82.6%), among whom 16 had cervical localization (84.2%). Hepatomegaly and/or splenomegaly affected 50% of patients. Twenty patients (90.9%) presented with digestive manifestations that were abdominal pain, diarrhea, or vomiting. One patient suffered from cholestasis and recurrent hyperammoniema episodes without other enzymatic deficiencies. The skin lesions were found in 19 patients (86.4%) that were mainly maculopapular rash. Nevertheless, urticarial lesions, and purpura were also reported. One woman experienced chronic cutaneous purpura persisting between attacks. Fourteen patients (63.6%) had pharyngitis, 13 patients (59.1%) experienced cough, and 6 patients (27.3%) suffered from otitis. Headache, aphtous ulcers, and ocular symptoms affected approximately one-third of patients. Three patients (13.0%) had conjunctivitis, 2 patients (9.1%) uveitis, and 1 patient (4.5%) optic neuritis. One patient had recurrent seizures related to MKD that began during adolescence. Psychiatric features, especially depression, were notified in 7 patients (31.8%). One patient was described as a schizoid personality. We identified 3 patients (13.6%) with renal involvement: segmental and focal glomerulosclerosis, systemic AA amyloidosis, and extracapillar glomerulonephritis. Two patients (9.1%) had renal angiomyolipoma. No macrophage activation syndrome was observed.

**TABLE 2:**
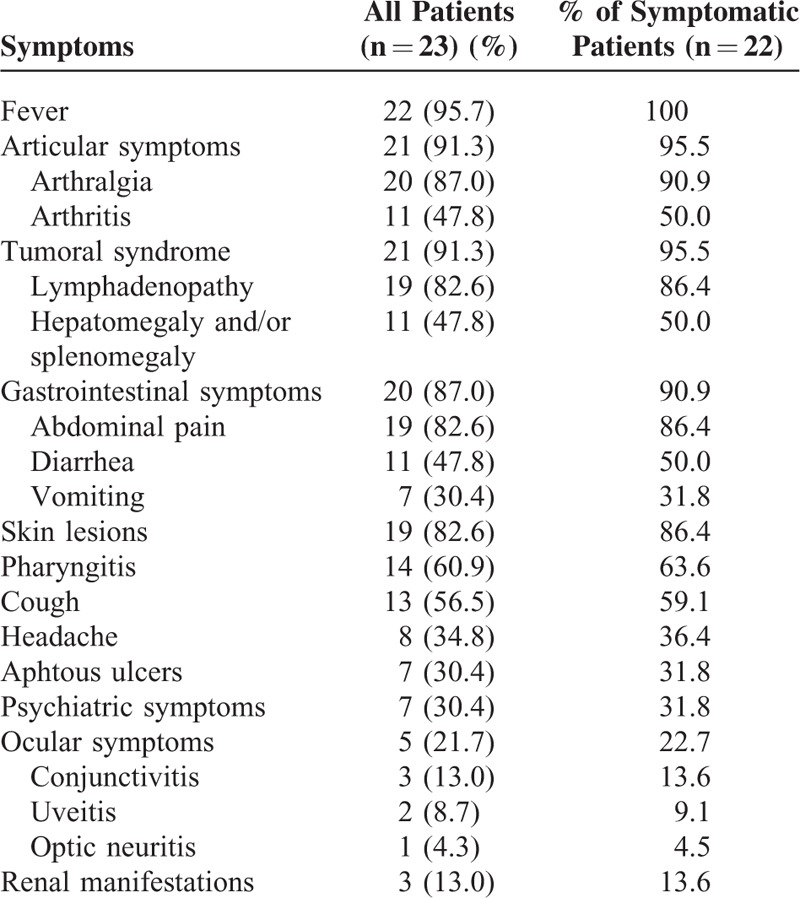
Clinical Symptoms During Attacks

### Laboratory Results

All patients had high elevated C-reactive protein (CRP) during attacks (median 24.1 mg/dL). All patients had microbiological analyses to exclude infectious diseases before considering inflammatory syndrome to be linked to MKD. Between febrile attacks, most of them had normalization of the inflammation markers, but 4 patients had low persistent biological inflammation (CRP ≤1 mg/dL). The biological results are shown in Table 3. An elevated IgD concentration was noted in 20 of the 21 tested patients (median 812 mg/L; range 330–3750); 1 patient had normal IgD concentration. The IgD dosage was high during febrile episode but also between febrile attacks. We used the highest known concentration of IgD to calculate the median and mean values of IgD values in our series. The IgA concentration was elevated to a median level of 7.6 g/L (range 3.8–16.9). The median IgG concentration was in normal range, and the median IgM level was decreased. The median MK activity was decreased to 0.1 μkat/kg protein (range 0–0.45). The median MA was elevated to 5.1 mmol/mol creatinine (range 1.7–28.2).

**TABLE 3:**
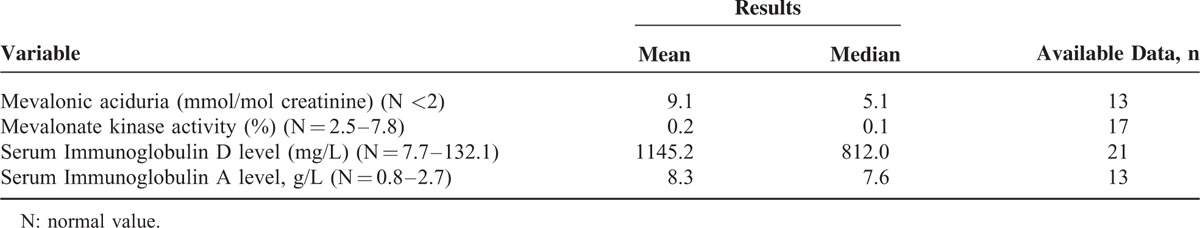
Laboratory Results

The MKD diagnosis was genetically confirmed in 21 patients (Table 4). Five patients were homozygous, and 16 were compound heterozygous. p.Val377Ile was the most prevalent mutation with a frequency of 40.5% of all MKD alleles, and was found in 14 of the 21 tested patients (66.7%). The 2 most common mutations (p.Val377Ile and p.Ile268Thr) accounted for 50% of mutations.

**TABLE 4:**
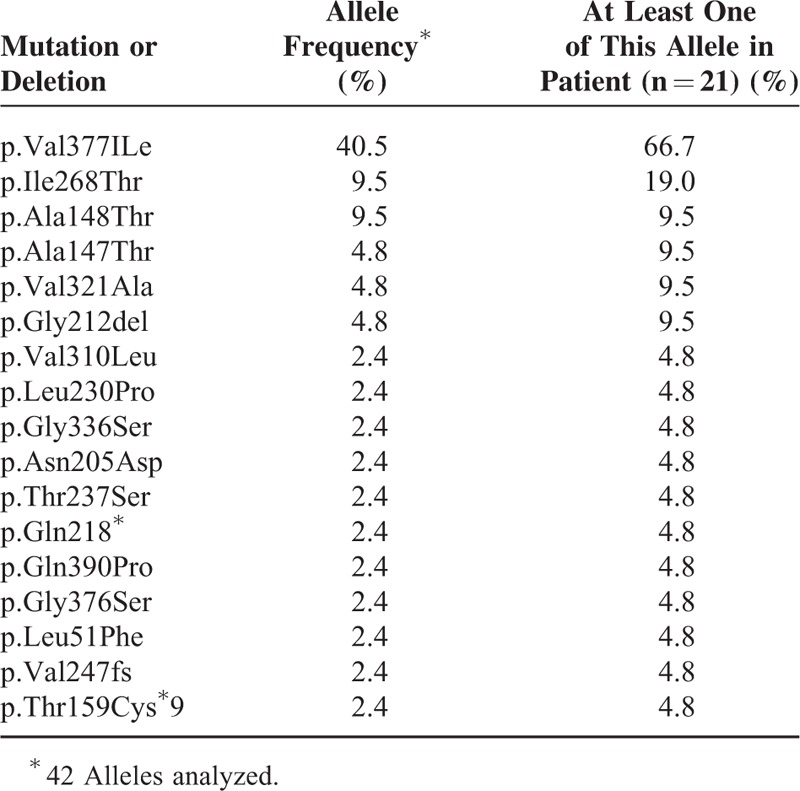
Mevalonate kinase mutations and allele frequencies

Nine patients experienced biopsies (lymphadenopathy, skin, bone marrow) that showed nonspecific neutrophilic inflammatory infiltrates.

### Treatments

Various treatments were tried to treat and prevent attacks (Table 5). Steroids were prescribed in 19 patients with partial to good response. Nonsteroidal anti-inflammatory therapies were used in 16 patients with an inconstant success. Colchicine (14 patients) aand statins (7 patients) were associated with limited or no response. Besides, statins were often bad-tolerated. Interleukin (IL)-1 receptor antagonists were used, with success, in 9 patients. Anti-tumor necrosis factor (TNF)α drugs (infliximab, adalimumab, etanercept) were used in 5 patients with partial response. Apart from those drugs, other molecules were administered with low or no success, and sometimes not well-tolerated: intravenous immunoglobulins, methotrexate, salazopyrine, hydroxychloroquine, leflunomide, azathioprine, thalidomide, mycophenolate mofetil, cyclosporine, rituximab, abatacept, tocilizumab, lenalidomide.

**TABLE 5:**
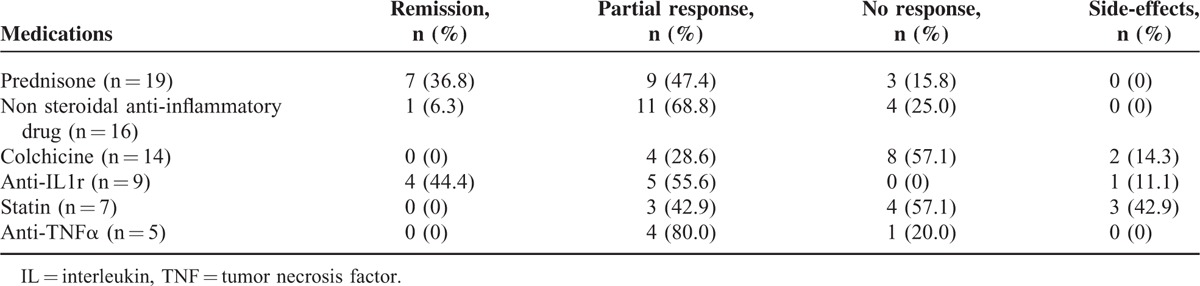
Treatments

### Follow-up

More than 65% of patients displayed a significant decrease in severity and frequency of attacks with increasing age, and in the absence of any immunomodulatory drug. Indeed, 12 patients among the 18 questioned patients attested a decrease in the number and the severity of the febrile attacks in adulthood. No data were available for 4 patients because of lost of follow-up. Only 7 patients (35%) achieved remission at the last follow-up visit; 5 of them received IL-1 receptor antagonist.

One woman developed AA amyloidosis that had revealed MKD, with renal and digestive localizations. She first developed proteinuria, then end-stage chronic kidney disease that required dialysis 7 years after MKD diagnosis. One patient had serious destructive arthritis altering quality of life.

Five patients (21.7%) died at a mean age of 56 years; 2 patients had septic shocks (one after digestive occlusion due to amyloidal colic stenosis), 2 had solid neoplasms (respectively liver and bladder tumor), and 1 suicide.

## DISCUSSION

This multicenter study reveals that the clinical and genetic spectrum of MKD diagnosed in adulthood does not furiously differ from classical pediatric phenotype. This observational study is, to our knowledge, the only series focusing on adulthood diagnosed MKD. Besides, it is the first to describe the natural history of the disease. The diagnosis delay could be linked to physician misunderstanding.

We confirm that MKD is a multisystemic disorder that could be associated with renal angiomyolipoma, erosive arthritis, crescentic glomerulonephritis, and neuropsychological disorders.^12,14^ Moreover, recurrent febrile attacks could lead to a severe complication, that is, AA amyloidosis as in 1 patient in our series. HIDS typical clinical features consist of high-grade fever episodes exceeding 40°C last 3 to 7 days and recurring every 4 to 6 weeks, sometimes triggered by vaccination, infection, trauma, or stress.^16^ A triggering factor is noted in 16 patients in our cohort (72.7%). Explanation should be a defective apoptosis of peripheral-blood lymphocytes leading to severe inflammatory response regarding on minor triggering factors.^17^ Pregnancy is suspected to trigger attacks in 3 patients in our series, but no physiopathological explanation is known. Reduced triggering events with increasing age may explain the lowest frequency and severity of attacks in adults.

Even if most patients have usually their first febrile attacks within the first year of life, the median age of diagnosis was 10 years with a median diagnosis delay of 9.9 years in the largest published series.^12^ In our cohort, >50% of patients are 5 years’ old or younger at onset of symptoms with a median age at onset of symptoms of 3 years, but a mean age of 9 years. The early childhood onset of febrile attacks is a key point of diagnosis; the criterion of onset before the age of 5 years is indeed a clinical feature that should evoke MKD.^18^ The latest age at onset of symptoms that we report could be explained by a memory bias. Indeed, adult patients self-report their own symptoms that could lead to imprecision. In pediatric studies, the histories of febrile attacks are reported by parents with more precision. However, 3 patients clearly described the beginning of febrile attacks during adolescence. In addition, this series confirms a significant diagnosis delay linked to medical misunderstanding of a rare disease. Moreover, frequent alternative diagnoses are retained before the good diagnosis. The median diagnosis delay in our series is 28.8 years. All these data support that MKD remains a diagnosis challenge, especially in adults.

As previously reported, arthralgia, lymphadenopathy, and abdominal symptoms are the most common organ involvements during attacks in our patients.^12,14^Table 6 shows MKD clinical features in the 2 largest previous series and in our cohort.^12,14^ Fever is constant. Arthralgia, abdominal pain, headache, and vomiting are significantly different between the 3 studies, but did not differ between our adulthood series, and the 2 previous series that included pediatric and adult patients. Compared with pediatric MKD, a latest diagnosis tends to lead to most common arthralgia and skin lesions, whereas diarrhea, vomiting, aphtous ulcers, and hepatosplenomegaly are less often reported (NS). There is a similar proportion of lymphadenopathy, abdominal pain, and arthritis. As already described, renal angiomyolipoma is observed in 2 patients. This large renal tumor leads to nephrectomy in one of our patients. The link between angiomyolipoma and MKD is not well explained, but our data and other cases series suggest that this association is not adventitious.^14^ In 1 case, MKD is diagnosed in a patient presenting with rapidly progressive crescentic glomerulonephritis; such manifestation has already been reported in MKD course in children.^14,19,20^ So, MKD should be evoked in patients presenting with crescentic glomerulonephritis without obvious diagnoses (lupus nephritis, ANCA-associated vasculitis, or Good-pasture disease) and a history of febrile attacks. Cholestasis observed in 1 patient has been previously reported.^21^ Psychiatric manifestations are not uncommon, mainly depressive conditions that could be related either to MKD clinical symptoms or owing to patients’ health status^12^ For instance, a new case of suicide occurred in 1 of our patients. Regarding neurological features, recurrent seizures occur in 1 patient. This may underline the continuous spectrum between HIDS and MA; no neurologic features are usually reported in HIDS.^6,18^ A chronic vasculitic purpura is described in 1 patient. Significantly, this purpura evolves individually without remission between attacks. We report cough as a frequent symptom during attacks. To our best knowledge, this is the first time that cough is described as a clinical feature of MKD. A feverish cough may mimic pulmonary infection and may delay diagnosis of MKD attacks and the corticosteroid introduction. Nevertheless, previous series suggested a susceptibility to bacterial infection in MKD.^12,14,22^ We report 2 deaths related to septic shock, meaning a link between immunodeficiency and MKD.^12,22,23^ However, the use of steroids could also explain a part of the infection susceptibility. Conversely to published data, we do not find hypogammaglobulinemia in our cohort.^14,24^

**TABLE 6:**
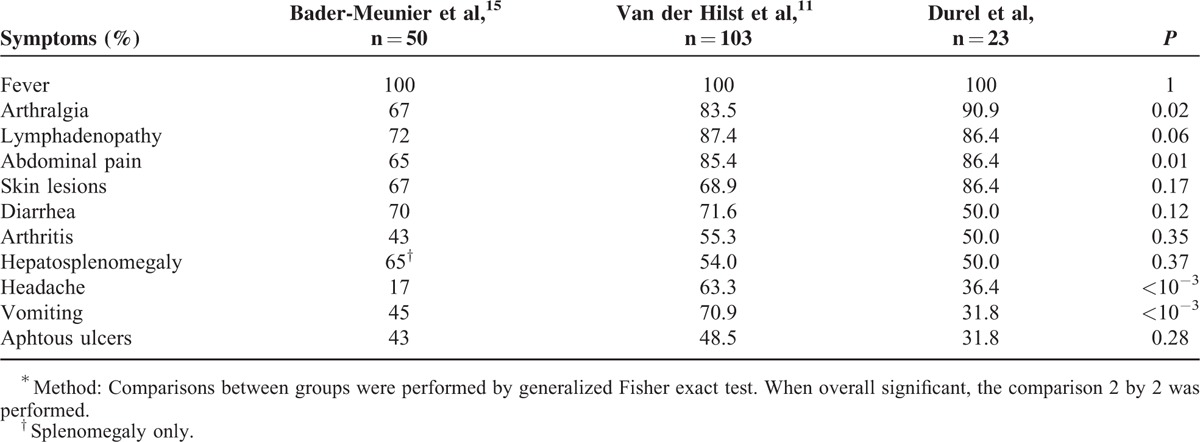
Comparison of Symptoms During Attacks Between the 2 Reference Previous Series and Our Cohort^∗^

The elevation of IgD has frequently helped guiding physicians to diagnose MKD in our series. Nevertheless, 1 patient exhibits normal IgD level what is concordant with precedent publications.^3,13,25,26^ Even if elevated IgD level is also found in other autoinflammatory diseases, strong elevation of IgD (>3 times the upper limit) is more specific for MKD. Our data illustrate the diagnostic value of the IgD dosage in adults compared with children (normal value in 4.3% in adults vs 22% in children).^12,13,27–29^ We observed persistent biological inflammatory syndrome between attacks in 4 patients. Surprisingly, the patient diagnosed with AA amyloidosis is not concerned. All of these patients have persistent febrile attacks despite anti-inflammatory drugs.

There is no obvious relation between phenotype and genotype. As already reported, the p.Val377Ile mutation is the most common mutation reported.^14^ All the mutations are registered in an online registry called INFEVERS.^30^ The 2 most prevalent mutations in our series are p.Val377Ile and p.Ile268Thr that is concordant with previous pediatric series.^12^ Two mutations (p.Leu230Pro and p.Val310Leu) have not been reported in children. Most of the others mutations represent only 1 allele in the cohort making difficult to conclude to any association between genetic status and specific clinical features. Furthermore, genotype-phenotype correlation studies in MKD are contradictory.^9,12,14,18,31^ Studies promote the hypothesis that there is no strict relationship between genotype and phenotype severity.^9,14^ p.Val377Ile is exclusively associated with the mild HIDS phenotype, whereas other mutations are found in both HIDS and MA, such as p.Ile268Thr.^31^ p.Val377Ile homozygous patients are attempted to have a milder severity of MKD or to be asymptomatic.^10^ In our series, the asymptomatic patient is p.Val377Ile homozygous, like her symptomatic sister with a mild activity of the disease. Nevertheless, one p.Val377Ile homozygous patient in our cohort exhibits a more serious HIDS phenotype. To explain the differences among the clinical spectrum of MKD, modulating factors should exist to attenuate the level of severity. Additional genetic studies should be performed to identify such potential modifying factors that could be mutations in introns, noncoding exons, squaring sequences or promoters in MK gene, or in another modifying gene. Some studies support the idea of a better correlation between clinical features and residual MK activity that would impact phenotype severity.^3,9,31,32^ MK activity is greatly affected in MA (<0.5% of normal) rather than in HIDS with an activity from 1.8% to 28%. In HIDS, most of the MK mutations affect stability or folding of the encoded mutant protein.^3^ In MA, mutations must have a more deleterious effect on MK protein folding and/or may direct effect MK catalytic properties.^31^ Besides, the residual activity of MK enzyme can be manipulated by environmental conditions; temperature worsens the folding defect.^31,33^ This must be clinically relevant in febrile patients, and could explain why ear-nose-throat (ENT) infection is a triggering factor of MKD attacks.

Considering treatments, most of our patients receive corticosteroids or nonsteroidal anti-inflammatory drugs. IL-1 receptor antagonist is used with success in 9 patients. Two prospective trials and others series confirm the effectiveness of anakinra in MKD.^14,34–37^ Further data are requested to draw definitive conclusions. This suggests a crucial role of IL-1 and inflammasome in the pathogenesis of MKD.^34,38^ Anakinra could be used daily or during febrile attacks only. Furthermore, a continuous treatment with anakinra decreases serum amyloid A level and could so prevent amyloidosis.^37^ Anakinra would have greater effect than anti-TNFα agents.^35^ Nevertheless, incomplete responses to anakinra suggest the implication of others cytokines.^39^ Advances in pathogenesis, that is, inflammasome activation, provide the basis for the development of new therapies. Successful treatment would prevent long-term severe complications.

Indeed, we report 2 infrequent but severe complications. The first case, already published, is a severe erosive arthritis in a young woman mimicking rheumatoid arthritis.^15^ There are serious structural damages with narrowing of metacarpophalangeal joint spaces and carpitis. Anakinra allows inflammatory joint pain resolution after failure of sulfasalazine, leflunomide, and etanercept. The second complication is a new case of systemic AA amyloidosis, revealing MKD. AA amyloidosis is a frequent and severe complication in the autoinflammatory diseases, but less common in HIDS (<3%).^40,41^ The usual decrease of MKD attacks with increasing age could explain the lowest risk of AA amyloidosis. Indeed, AA amyloidosis complicates disorders with sustained acute-phase response and persistent elevation of serum amyloid A protein level.^42^ Unfortunately, we were not able to report the dosage of serum amyloid A protein in our patients. To our knowledge, 5 previous cases of AA amyloidosis in MKD have been published.^43–46^ All cases have histories of febrile attacks since childhood, and are 7 to 29 years’ old. We report the sixth case. The patient first develops nephrotic syndrome, then end-stage chronic kidney disease, and is finally dialyzed at 69 years. Histories of recurrent febrile attacks have led to MKD diagnosis at 70 years. She suffers from febrile crises for 60 years before onset of an overt proteinuria. She is compound heterozygous (p.Val377Ile - p.Ile268Thr) sharing the same genetic status with another of the 5 patients.^45^ Despite lower frequency of AA amyloidosis in MKD, such a condition could complicate long-term course of the disease.^13,47^ Early diagnosis and treatment of MKD are needed to prevent development of the life-threatening systemic AA amyloidosis.

To conclude, this study has some limits related to its retrospective character leading to a potential lack of data, and memory bias. We try to limit bias by contacting each referring physicians; we collect a maximum of written information to ameliorate exhaustivity. Besides, we exclude from analysis 3 patients because of insufficient data. All but 2 cases are genetically proven and systematically collect from laboratories of interest. This is the first study interesting in adulthood MKD and reporting about the spontaneous course of the disease. MKD diagnosed in adulthood shares clinical and genetic features with classical pediatric disease (see Table 6). Interestingly, we report a good diagnostic value of the serum IgD in adults. In cases of suspicion of autoinflammatory disease in adults, we suggest to dose IgD to help physicians to diagnose MKD. Despite the dramatic symptoms during febrile attacks and alteration of quality of life, prognosis remains good. Despite decrease of the severity and frequency of attacks with increasing age, only one-third of patients achieve remission. Amyloidosis AA is rare but could complicate long-term course of MKD. Physicians must keep in mind the existence of yet undiagnosed MKD in adults because diagnosis and initiation of a successful treatment would prevent febrile attacks and their complications, and improve quality of life.
